# Cancers in Agreement? Exploring the Cross-Talk of Cancer Metabolomic and Transcriptomic Landscapes Using Publicly Available Data

**DOI:** 10.3390/cancers13030393

**Published:** 2021-01-21

**Authors:** Derek van Tilborg, Edoardo Saccenti

**Affiliations:** Laboratory of Systems and Synthetic Biology, Wageningen University & Research, Stippeneng, 6708 WE Wageningen, The Netherlands; derek.vantilborg@wur.nl

**Keywords:** association networks, cancer metabolism, biological networks analysis, gaussian graphical models, pathway analysis

## Abstract

**Simple Summary:**

Changes in metabolism are a well-known characteristic of cancer cells. Different cancer types are unique in their genetic aspects, but also in their metabolism, which is in turn, governed by genetics. The aim of our study was to find these differences in metabolic behavior across different cancer types and uncovering intersections between gene expression and metabolic deregulations. We scoured the public domain for metabolomics and transcriptomics data from clinical profiling studies to perform a comprehensive comparison study. By combining evidence from both the genetic and the metabolic aspects, we described the most prominently aberrated pathways across eight different cancer types together with their metabolomic and transcriptomics similarities.

**Abstract:**

One of the major hallmarks of cancer is the derailment of a cell’s metabolism. The multifaceted nature of cancer and different cancer types is transduced by both its transcriptomic and metabolomic landscapes. In this study, we re-purposed the publicly available transcriptomic and metabolomics data of eight cancer types (breast, lung, gastric, renal, liver, colorectal, prostate, and multiple myeloma) to find and investigate differences and commonalities on a pathway level among different cancer types. Topological analysis of inferred graphical Gaussian association networks showed that cancer was strongly defined in genetic networks, but not in metabolic networks. Using different statistical approaches to find significant differences between cancer and control cases, we highlighted the difficulties of high-level data-merging and in using statistical association networks. Cancer transcriptomics and metabolomics and landscapes were characterized by changed macro-molecule production, however, only major metabolic deregulations with highly impacted pathways were found in liver cancer. Cell cycle was enriched in breast, liver, and colorectal cancer, while breast and lung cancer were distinguished by highly enriched oncogene signaling pathways. A strong inflammatory response was observed in lung cancer and, to some extent, renal cancer. This study highlights the necessity of combining different omics levels to obtain a better description of cancer characteristics.

## 1. Introduction

The vast amount of publicly available multi-omics databases with associated clinical annotation including tumor histology, patient response, and outcome is enabling a multidimensional approach to cancer investigations [1].

Among omics disciplines, metabolomics and transcriptomics have been favored by systems biology tools to explore cancer biology. Metabolomics, i.e., the study of the nature and the concentration profiles present in biofluids and tissues [2], has been usually deployed in the clinical setting to investigate possible diagnostic or prognostic biomarkers and to monitor patients [3,4,5], rather than to unravel cancer metabolism mechanisms or comparing differences between cancers. This is due to the reactive and circumstantial nature of metabolism, even the most advanced measuring platforms can only take a snapshot of the whole metabolome [6]. Combining multiple snapshots poses many technical difficulties, including differences between measuring platforms, batch effects, and metabolite identification.

In contrast to metabolomics, transcriptomics is a more holistic and standardized approach where almost all gene transcripts can be measured [7]. This has led to the identification of biologically important genes and pathways frequently disrupted across many cancer types and has revealed clinically relevant diagnostic, prognostic, and druggable targets [8,9].

However, studies at different levels have clearly indicated that the complex nature of cancer cannot be fully captured considering only one *omics* level at a time [10,11,12] and that defining cancer hallmarks requires the characterization of molecular alterations at multiple levels [10].

Using an integrative approach, we re-purposed publicly available metabolomics data created for biomarker-discovery in combination with gene expression data to find differences and commonalities between eight cancer types at the biological pathway level. We considered eight different cancer types (breast, lung, gastric, renal, liver, colorectal, prostate, and multiple myeloma) and we explored and compared the associated metabolomic and transcriptomic landscapes.

We combined standard univariate differential metabolite abundance and gene expression analysis with the inference and analysis of metabolite and gene association networks, since relevant information is contained in the relationships among molecular features and not in levels only [13,14].

We aggregated results from the metabolomic and transcriptomic levels at the pathway level to overcome the problem of integrating data from different studies. We showed fundamental differences between the metabolomic and transcriptomic landscape of different cancer types and highlighted that a better description of cancer characteristics is obtained when different omics levels are considered.

## 2. Results and Discussion

### 2.1. Data Collection

We collected 14 metabolomics data sets encompassing eight different cancer types (breast, lung, gastric, renal, liver, colorectal, prostate, and multiple myeloma from organ tissue (5), urine (2), blood plasma (4) and serum (3)) that satisfied our inclusion criteria. Eight transcriptomics data sets, matching the same cancer type of the metabolomics data sets were also obtained. A complete description of all data sets can be found in Table 1. Data collection is described in more detail in Appendix A. Metabolite occurrence across all data sets is given in Table S1.

### 2.2. Analysis of Cancer Metabolomics Data

#### 2.2.1. Differentially Abundant Metabolites in Different Cancer Types

We compared metabolite abundances between cancer and controls samples for each cancer type (at the data set level) to investigate shared and different metabolic features across different types of cancer and/or tissues. It proved hard to replicate the findings reported in the original papers because of different (and often sub-optimal: lack of data transformation, adjustment for multiple testing) data-analysis strategies and no paper could be exactly replicated.

For six data sets (corresponding to renal, prostate, gastric, breast, and lung cancer) we found none or less than five significantly different abundant metabolites between case and controls; the remaining data sets gave varying numbers of differentially abundant metabolites as shown in Table 2. Out of the 459 significant differentially abundant metabolites, 408 metabolites were found to be specific to only one cancer type.

The most common differentially abundant metabolites are taurine, proline, and glutamic acid, found in four out of the seven remaining cancer types, confirming the role of these amino acids [24,25,26,27,28] and of amino acids in general in cancer biology [29].

We found lactic acid to be differentially abundant in breast, liver, and lung cancer. Lactic acid is a well-known marker for the Warburg effect [30] indicating the use of glycolysis in cancer cells to shuttle more resources towards gaining cell mass. There is ample literature about lactic acid either as a marker of tumor activity or regulator [31,32,33].

These results are in line with the findings of a recent meta-analysis by Goveia et al. [34] which also found lactic acid, glutamic acid, tryptophan, histidine, glutamine, and kynurenine to be often reported in more than 200 cancer metabolomics studies. However, it should be noted that they used a vote-counting approach to compare the reported findings of multiple studies rather than re-analyzing data like in our study.

We used Pathway Enrichment Analysis on the cancer-specific differentially abundant metabolites to reduce results to a smaller and more interpretable set of altered processes [35] (all pathway analysis results are available in the Supplementary File Pathway.zip).

We took into account how much each pathway is impacted (i.e., its relevance) by the selected metabolites since not all metabolites have the same relevance to defining the overall pathway structure (see Methods) and not all metabolic pathways are relevant for different cancer types [36].

**Figure 1:**
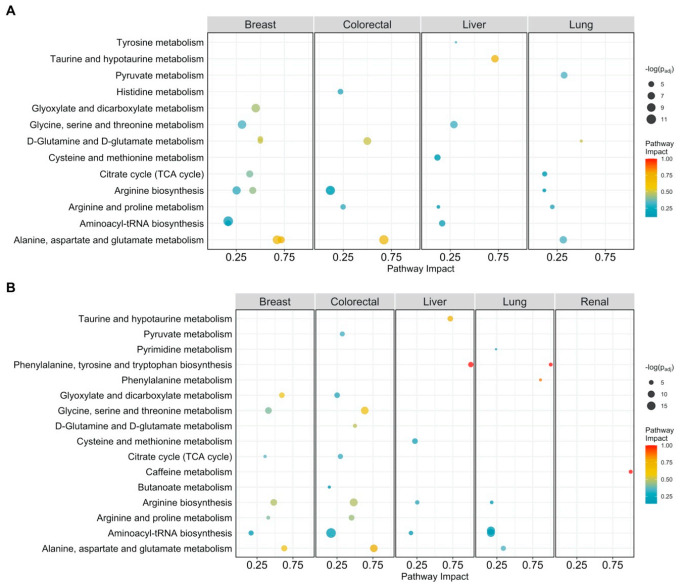
Pathway analysis plot showing the impact on metabolic pathways that are significantly enriched (*p_adj_ < 0.05*) and have a pathway impact score > 0.1 per cancer type. Datapoints are colored according to their impact score. (**A**) Pathway impact according to differentially abundant metabolites (*p_adj_ < 0.05*); (**B**) pathway impact according to differentially connected metabolites (Benjamini-Hochberg adjusted *p*-value *p_adj_* < 005).

Overall, we found 18 unique enriched pathways in breast, liver, lung, and colorectal cancer, while no enriched pathways were found in multiple myeloma, as shown in Figure 1A. The most commonly enriched pathway between cancer types, aminoacyl-tRNA biosynthesis, is but of little relevance given its moderate impact. The highly impacted pathways are (*i*) glycine, serine, and threonine metabolism in breast and liver cancer, (*ii*) taurine and hypotaurine metabolism, and glyoxylate and dicarboxylate metabolism in liver and breast cancer, respectively, and (*iii*) D-glutamine and D-glutamate metabolism, and arginine biosynthesis in colorectal, breast, and lung cancer.

Serine and glycine are biosynthetically linked and are precursors for the synthesis of proteins, nucleic acids, and lipids that are crucial to cancer cell growth [37,38,39]. The taurine and hypotaurine metabolic pathway has been shown to be relevant to multiple types of cancers, such as ovarian, lung, colon, and renal cancers, and was recently associated with breast cancer [15]. These data have been re-analyzed in the present study, but we did not find this pathway to be enriched in breast cancer but only in liver cancer. To the best of our knowledge, this association has never been reported.

**Figure 2:**
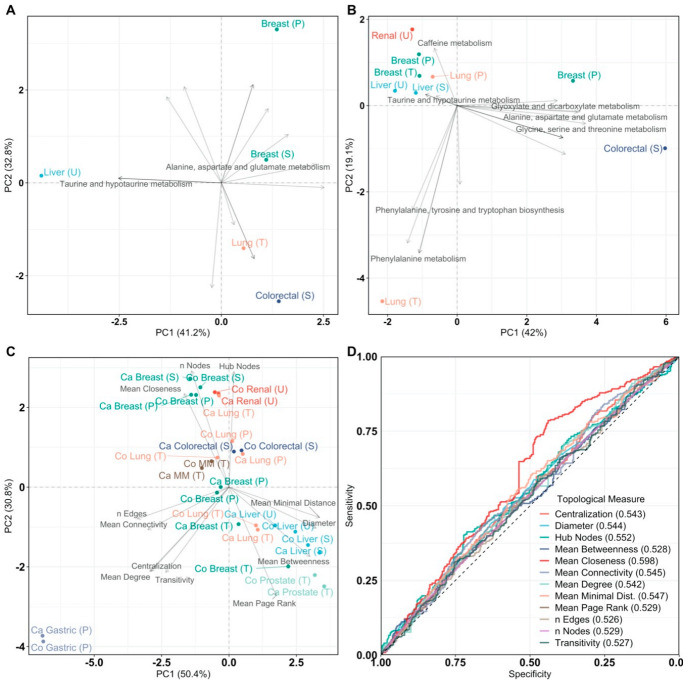
Principal Component Analysis (PCA) of pathway impact and network analysis. PCA labels are colored according to their cancer type and PCA loadings are labeled if they have a pathway impact score > 0.5. Sample origin is labeled as T, U, S, P for tissue, urine, serum, and plasma, respectively. Multiple myeloma is abbreviated as MM. (**A**) PCA biplot of pathway impact based on differentially abundant metabolites per cancer type; (**B**) PCA biplot of pathway impact based on differentially connected metabolites per cancer type; (**C**) PCA biplot of topological network measures per cancer type for all inferred metabolite association networks. Here, networks inferred from cancer or control samples are labeled as “Ca” and “Co”, respectively; (**D**) receiver operating characteristic curves of topological measures predicting cancer pathology on metabolite association networks (n = 28, 14/14). Predictions are based on logistic regression, cross-validated with leave-one-out cross-validation. The Area Under the Curve (AUC), 95% confidence interval (CI), and *p*-value of all topological measures are Nodes: AUC = 0.529, 95% CI = 0.488–0.570, *p* = 0.170, Edges: AUC = 0.526, 95% CI = 0.485–0.567, *p* = 0.216, Mean Connectivity: AUC = 0.545, 95% CI = 0.504–0.586, *p* = 0.0302, Mean Degree: AUC = 0.542, 95% CI = 0.501–0.583, *p* = 0.0470, Mean Closeness: AUC = 0.598, 95% CI = 0.557–0.638, *p* = 2.20 × 10^−6^, Mean Betweenness: AUC = 0.528, 95% CI = 0.487–0.570, *p* = 0.175, Diameter: AUC = 0.544, 95% CI = 0.503–0.585, *p* = 0.0369, Mean Minimal Distance: AUC = 0.547, 95% CI = 0.506–0.588, *p* = 0.0244, Mean Page Rank: AUC = 0.529, 95% CI = 0.488–0.570, *p* = 0.165, Hub Nodes: AUC = 0.552, 95% CI = 0.511–0.593, *p* = 0.0137, Centralization: AUC = 0.543, 95% CI = 0.502–0.584, *p* = 0.0422, and Transitivity: AUC = 0.527, 95% CI = 0.486–0.568, *p* = 0.196. Definitions of topological measures are given in Methods.

We applied Principal Component Analysis (PCA) on an n_cancer_ × n_pathway_ matrix containing pathway impact scores, to comprehensively evaluate pathway enrichment analysis and to highlight the relationships at the metabolic level among different cancer types. Figure 2A shows how different cancer types are characterized by the alteration of different metabolic pathways. PCA (Figure 2A) shows a large separation between all cancer types. Liver cancer is characterized by tyrosine metabolism, taurine, and hypotaurine metabolism, and cysteine and methionine metabolism, while lung cancer is characterized by glycine, serine, and threonine metabolism, which is also an attribute of one of the two breast cancers (data set ST000355).

#### 2.2.2. Cancer-Specific Metabolite–Metabolite Association Networks

We took a systems-based approach by inferring statistical metabolite–metabolite association networks [14] (see Methods). Networks and pathways are similar concepts but bear different and complementary information [35]. Pathways are small-scale systems of well-studied processes while (association) networks comprise system-wide association among molecular features (in our case metabolites) resulting in simplified abstractions of complex biological phenomena and are likely to contain novel information not covered in well-defined pathways [35].

In total, we obtained 14 networks specific to eight different cancer types and 14 matched control networks. We explored network topology by considering a set of standard topology measures that can be used to summarize the network characteristics [40,41], which are given in Table 3.

To compare comprehensively the cancer-specific metabolite–metabolite association networks with respect to their topological features and sample type (cancer or healthy control), we performed PCA on the data given in Table 3. The resulting biplot is given in Figure 2C. PCA showed that cancer networks are more similar to their control networks than to other cancer-specific networks. Besides, different control networks inferred from the same origin (tissue, urine, or blood) are highly different in network topology. It can be observed that different control networks inferred from the same origin (tissue, urine, or blood) have highly different network topologies as well.

Given the low separation between cancer and control networks in the PCA, we performed logistic regression to quantify the association between the topological measures and cancer status, i.e., comparing the topology measures of cancer and control networks. The Receiver-Operator Curves (ROC) for these models are shown in Figure 2D. For all topological measures, the Area Under Curve (AUC) is around 0.5 and the highest achieved is 0.598 for the mean closeness. This indicates that measures describing overall network topology are weak estimators to discriminate between cancer and health-specific metabolite–metabolite association networks. In other words, the characteristics of metabolic association networks of cancer cells are almost non-distinguishable from normal cases. Overall, these results indicate that differences able to discriminate between metabolite–metabolite association networks specific to cancer and those specific to a healthy state must be looked for at the metabolite/node level through differential network analysis which is presented in the following section.

#### 2.2.3. Differential Metabolite–Metabolite Network Analysis

To investigate differences of metabolite–metabolite association networks specific to cancer and health status at the metabolite level, we performed differential connectivity analysis. In total, we found 1099 statistically significant (*p*-value adjusted < 0.05) differentially connected metabolites across data sets or 925 across cancer types. Of these metabolites, 646 were unique, furthermore, 486 were found only once in any of the seven cancer types. The most common metabolites were glutamine, kynurenine, leucine, phenylalanine, pyruvic acid, and sorbitol, which occurred in five out of seven cancer types. An overview of the number of differentially connected metabolites per cancer type is given in Table 4.

Similar to what was done in the case of differentially abundant metabolites, we performed pathway impact analysis on the differentially connected metabolites and found 21 significantly enriched pathways: aminoacyl-tRNA biosynthesis and arginine biosynthesis were enriched in four out of five cancer types. Figure 1B shows the impact of each pathway. The most impacted are taurine and hypotaurine metabolism in liver cancer, phenylalanine, tyrosine, and tryptophan biosynthesis, in liver and lung cancer, and phenylalanine metabolism in lung cancer. Deregulations of tyrosine metabolism in hepatocellular carcinoma have been reported in the literature as well [42,43]. Hypotaurine has been shown to activate hypoxia signaling in vitro [44], which is an important survival strategy of cancer cells. Multiple amino-acid related pathways like glycine, serine, and threonine metabolism, and alanine, aspartate, and glutamate metabolism are highly impacted in breast and colorectal cancer.

Figure 2B shows the PCA plot on the pathway impact. We observed that colorectal cancer and one of the three cases of breast cancer (data set ST000355) are characterized by a highly impacted glycine, serine, and threonine metabolism, while also having their alanine, aspartate, and glutamate metabolism impacted. Glutamate serves a critical role in regulating the signaling kinases MEK (mitogen-activated protein kinase) and ERK (extracellular signal *regulated* kinase) [45,46]. Besides, glutamate is closely connected to the TCA (tricarboxylic acid) cycle [47]. Both cancer types are also found to be directly affected by the citrate cycle and glyoxalase and dicarboxylate metabolism, which are biochemically tightly linked. This suggests that in breast and colorectal cancer, the citrate cycle is highly affected. Lung cancer tissue is the only cancer type that is differentiated from all other types by its phenylalanine metabolism, pyrimidine metabolism, and pantothenate and CoA biosynthesis.

It is interesting to note how in the case of lung and breast cancer, different metabolic signatures are obtained when considering metabolites derived from tissue or blood, while for liver cancer differences in urine and blood point to the same altered pathways. This complicates the definition of a single hallmark for every cancer type and highlights the necessity for investigation at different levels.

These results, combined with the analysis of differentially expressed metabolites, indicate the existence of a pan-cancer metabolic fingerprint characterized by altered protein metabolism.

### 2.3. Analysis of Cancer Transcriptomics Data

#### 2.3.1. Differentially Expressed Genes

We calculated differentially expressed genes by comparing expression levels between cancer and control tissue samples in eight transcriptomics data sets that matched to the metabolomics data in cancer type. Twenty-five differentially expressed genes were shared across seven cancer types. Most genes, however, were not shared among the majority of cancer types: 6251 genes were only significantly expressed in one cancer type. Results are summarized in Table 5.

We performed Gene Ontology (GO) enrichment on the sets of differentially expressed genes focusing on “biological process” ontology, which accounts for changes on the level of granularity of the cell that is mediated by one or more gene products [48]. The 10 most significantly enriched terms per cancer type were mostly related to the cell cycle pathways. These pathways involve well-orchestrated transcriptional and epigenetic controls regulating the cell division process [49]. Breast, liver, and colorectal cancer were mostly enriched in cell cycle-related terms, and renal cancer was characterized by a deregulated TCA cycle. Prostate and lung cancer were enriched for cellular organization and morphogenesis related terms, while gastric, colorectal cancer, and multiple myeloma, on the other hand, showed to favor RNA and protein synthesis-related GO terms.

**Figure 3:**
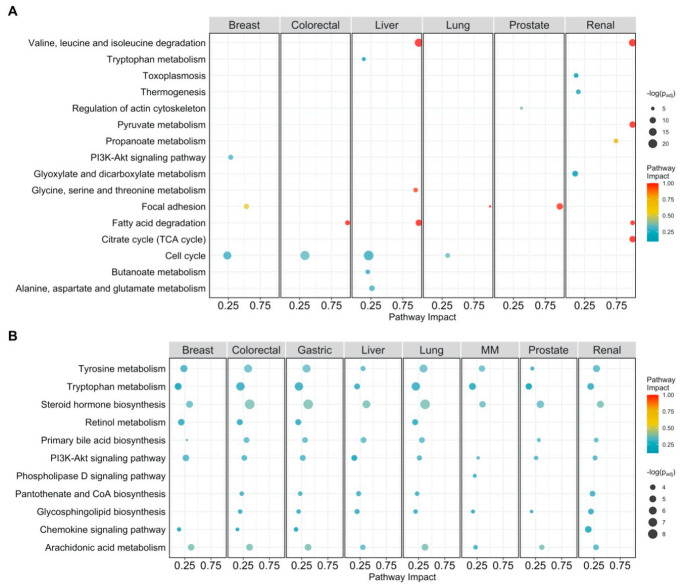
Pathway analysis plot showing the impact on cellular pathways that are significantly enriched (*p_adj_ < 0.05*) and have a pathway impact score > 0.1 per cancer type. Datapoints are colored according to their impact score. Multiple myeloma is abbreviated as MM. (**A**) Pathway impact according to differentially expressed genes (*p_adj_ < 0.05*); (**B**) pathway impact according to differentially connected genes (*p_adj_ < 0.05*). *p_adj_* indicates Benjamini–Hochberg corrected *p*-values.

We performed a pathway impact analysis using the significantly expressed genes from the eight cancer types. An overview of the results of this analysis can be found in Figure 3A. 

In agreement with GO enrichment analysis, we found the most commonly impacted pathway across cancer types to be the cell cycle. This pathway is enriched in breast, liver, colorectal, and lung cancer. The second most common pathway is fatty acid degradation, which is enriched in colorectal, liver, and renal cancer. None of these results are surprising: dysregulation of the cell cycle can lead to various diseases, including cancer [50,51,52], and reprogramming of fatty acid metabolism in cancer has been discussed as a means to sustain the production of ATP and macromolecules needed for cell growth, division and survival [53,54,55].

We observed several low impacted pathways like disease and pathogen-related pathways to be enriched in renal [56,57], and breast [58] cancer. Breast cancer is the only cancer type that showed enrichment in a number of low-impact cancer-specific pathways and an oncogene pathway (PI3K-Akt signaling [59]), suggesting this kind of cancer to be especially driven by a high number of prominent oncogenes.

The focal adhesion (FA) pathway (FAs are large protein complexes that connect the cell cytoskeleton to the ECM through integrins [60]) is highly impacted in breast, lung, and prostate cancer. The latter being enriched in GO terms related to cell morphology, cell adhesion, and the actin cytoskeleton. Cancer cells exhibit highly altered focal adhesion dynamics [60,61] and cell adhesion, and matrix stiffness plays a pivotal role in cancer cell invasion and metastasis [62,63].

Consistent with the observation that changes in metabolism are a well-known characteristic of cancer cells [64,65], we observed numerous highly impacted pathways related to metabolism. However, different cancer types were impacted by different metabolic pathways.

In liver cancer, we found alanine, aspartate, and glutamate metabolism, glycine serine, and threonine metabolism, butanoate metabolism, tryptophan metabolism, and fatty acid degradation to be highly impacted, while we observed valine, leucine, and isoleucine degradation in the liver and in renal cancer as well. This latter pathway is used to generate intermediates that can later be used for macromolecule synthesis [66,67].

**Figure 4:**
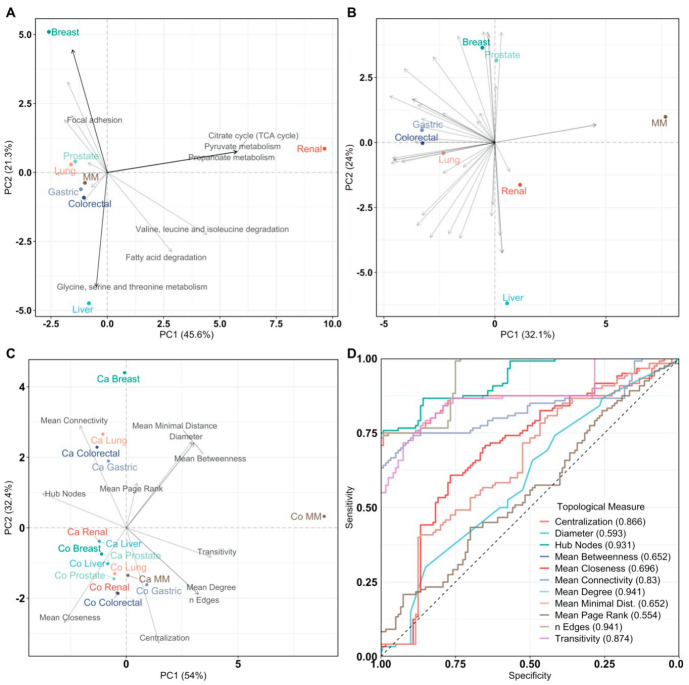
Principal Component Analysis of pathway impact and network analysis. PCA labels are colored according to their cancer type and PCA loadings are labeled if they have a pathway impact score > 0.5. Multiple myeloma is abbreviated as MM. (**A**) PCA biplot of pathway impact based on differentially abundant genes per cancer type; (**B**) PCA biplot of pathway impact based on differentially connected genes per cancer type; (**C**) PCA biplot of topological network measures per cancer type for all inferred gene networks. Here, networks inferred from cancer or control samples are labeled as “Ca” and “Co”, respectively; (**D**) receiver operating characteristic curves of topological measures predicting cancer pathology on gene association networks (n = 16, 8/8). Predictions are based on logistic regression, cross-validated with leave-one-out cross-validation. The Area Under the Curve (AUC), 95% confidence interval (CI), and *p*-value of all topological measures are Edges: AUC = 0.941, 95% CI = 0.915–0.967, *p* = 7.59 × 10^−242^, Mean Connectivity: AUC = 0.830, 95% CI = 0.774–0.886, *p* = 8.80 × 10^−31^, Mean Degree: AUC =0.941, 95% CI = 0.915–0.967, *p* = 7.59 × 10^−242^, Mean Closeness: AUC = 0.696, 95% CI = 0.628–0.764, *p* = 1.85 × 10^−8^, Mean Betweenness: AUC = 0.652, 95% CI = 0.582–0.722, *p* = 2.14 × 10^−5^, Diameter: AUC = 0.593, 95% CI = 0.521–0.665, *p* = 0.0114, Mean Minimal Distance: AUC = 0.652, 95% CI = 0.582–0.722, *p* = 2.14 × 10^−5^, Mean Page Rank: AUC = 0.554, 95% CI = 0.481–0.627, *p* = 0.146, Hub Nodes: AUC = 0.931, 95% CI = 0.902–0.961, *p* = 2.93 × 10^−178^, Centralization: AUC = 0.866, 95% CI = 0.813–0.919, *p* = 8.00 × 10^−42^, and Transitivity: AUC = 0.874, 95% CI = 0.828–0.92, *p* = 1.48 × 10^−56^. Definitions of topological measures are given in Methods.

Renal cancer was enriched for propanoate metabolism, glyoxylate and dicarboxylate metabolism, pyruvate metabolism, oxidative phosphorylation, and the TCA cycle. Mutations in genes related to the TCA-cycle enzymes succinate dehydrogenase and fumarate hydratase have been associated with renal cancer and correlated with the development of renal tumors [68,69,70]. PCA (see Figure 4A) was performed on an n_cancer_ × n_pathway_ matrix containing pathway impact scores, to summarize and visualize cancer (dis)similarities. We found that renal cancer is characterized by deregulation of TCA cycle-related pathways, pyruvate metabolism, propanoate metabolism, apoptosis, and its pathogenetic signature. Breast cancer, on the other hand, was characterized by cancer-specific pathways and cellular senescence pathway. Liver cancer was mostly distinguished by its metabolic character: tryptophan metabolism, glycine, serine, and threonine metabolism, alanine, aspartate, and glutamate metabolism, and butanoate metabolism.

#### 2.3.2. Cancer-Specific Gene–Gene Association Networks

Similarly, to what was done with metabolite data, we inferred gene–gene association networks to investigate gene relationships and their association with cancer types. Because of the computational burden, we restrained our analysis to genes with sigma >1 across the different data sets. In total, we inferred 16 matched networks for cancer and control groups encompassing 2570 genes. We characterized the gene–gene networks using several topological measures (see Table 6) and we observed, by applying PCA, a large separation between all individual cancer types and their corresponding control network (see Figure 4C).

Applying logistic regression on the topological measures, we could discriminate between cancer- and control-specific networks as shown by the ROC curves in Figure 4D: the number of edges, the mean degree, and the number of hub nodes in a network gave an area under the curve of 0.941, 0.941, and 0.931, respectively. These best predictors indicate that cancer-associated genes exhibit less interdependence with other genes than in normal genes with some genes becoming an important genetic hub: this observation is consistent with the notion of cancer driver genes [71].

A similar exercise was attempted by Ramadan et al. [72], and our results confirm that the organization of genetic codependences is different between cancer and normal cells and thus can be used to discriminate between the two statuses. However, as shown in a previous section, the same cannot be achieved using the topology of metabolite–metabolite association networks.

#### 2.3.3. Differential Network Analysis

By means of differential connectivity analysis, we selected genes that showed differential connectivity between controls and cancers, most of which are also differentially expressed, as shown in Table 7. GO enrichment of this gene set yielded terms associated with mitotic cell cycle process, cell cycle process, cellular amide metabolic process, immune effector process, peptide metabolic process, and mitotic cell cycle in multiple myeloma, and the GO term regulation of macromolecule metabolic process in breast cancer.

Subsequently, pathway impact analysis found many of the same pathways to be enriched in all cancer types, as can be seen in Figure 3B. This gene list may be biased because of the gene pre-selection, however, PCA (Figure 4B) on pathway impact scores shows that cancer types are still separated from each other. Breast and prostate cancer are mostly defined by the PI3K-Akt signaling pathway. Multiple myeloma is separated by its impacted phospholipase D signaling. Liver cancer, on the other hand, is defined by gastric acid secretion function, a pathway that was not highlighted in the analysis of differentially expressed genes and that has been reported in the literature [73].

### 2.4. Joint Pathway Analysis

Cellular pathways and reaction networks are not controlled by metabolites or genes alone, but by a complex interplay between genetics and metabolism [74]: therefore, we integrated the results from metabolite and gene differential abundance/expression and connectivity analysis to perform a joint pathway analysis using the approach proposed by Chong et al. [75].

#### 2.4.1. Joint Pathway Analysis of Differentially Expressed Genes and Differentially Abundant Metabolites

**Figure 5:**
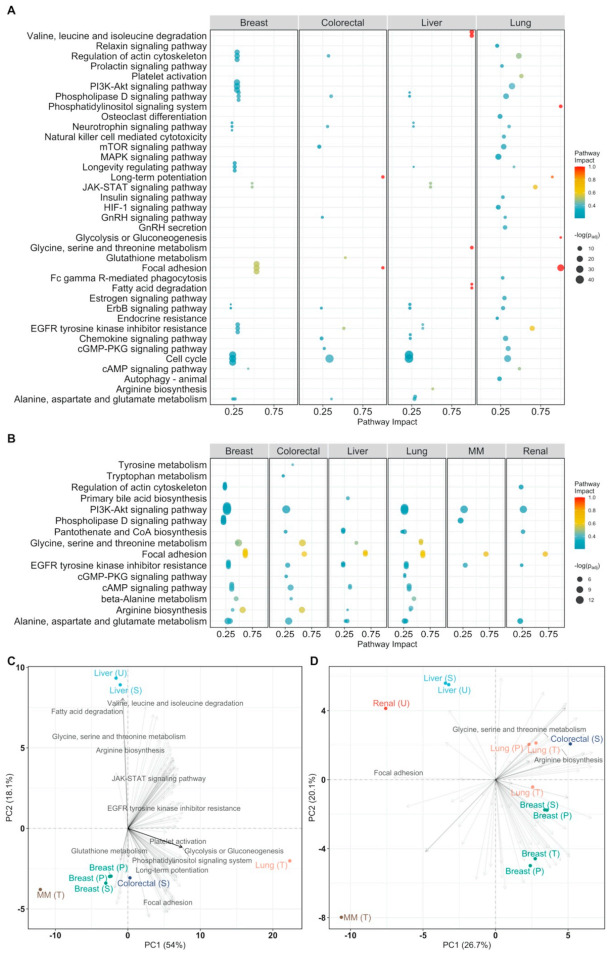
Joint pathway analysis. Multiple myeloma is abbreviated as MM. Sample origin of the metabolomics counterpart is labeled as T, U, S, P for tissue, urine, serum, and plasma, respectively. (**A**) Pathway analysis score plot of the impact on cellular pathways that are significantly enriched (*p_adj_* < 0.05) per cancer type according to differentially expressed genes (*p_adj_* < 0.05) and differentially abundant metabolites (*p_adj_* < 0.05); (**B**) pathway analysis score plot of the impact on cellular pathways that are significantly enriched (*p_adj_ <* 0.05) per cancer type according to differentially connected genes (*p_adj_ <* 0.05) and differentially connected metabolites (*p_adj_ <* 0.05). Data points are colored according to their impact score; (**C**) PCA biplot of pathway impact based on differentially expressed genes and differentially abundant metabolites per cancer type; (**D**) PCA biplot of pathway impact based on differentially connected genes and differentially connected metabolites per cancer type. Datapoints are colored according to their cancer type. PCA loadings are labeled only if corresponding to a pathway impact score >0.5. *p_adj_* indicates Benjamini–Hochberg corrected *p*-values.

In breast, liver, colorectal, lung cancer, and multiple myeloma, we found enough significantly changed metabolites and genes to perform joint pathway analysis, which is summarized in Figure 5A: in total, 149 different pathways were significantly enriched.

From the PCA shown in Figure 5C, we observed a good separation between most different cancer types, with colorectal, breast, liver, and multiple myeloma clustering together. We also observed that in this case, both liver data sets and all three breast data sets cluster together as well. As this was not the case in the separate metabolite or gene-based analyses (see Figure 2 and Figure 4), this confirms that the genetic and metabolic aspect alone is not enough for a full characterization of cancer similarities and differences.

Overall, we observed that lung cancer seems to possess a particular metabolic signature and it is well separated from the other cancer types. Multiple myeloma yielded only one significantly enriched pathway: the ribosome pathway while the other cancers all were enriched for cell cycle, DNA replication, Human T-cell leukemia virus 1 infection, and the more general pathways in cancer pathway. In liver cancer, enrichment of arginine biosynthesis, and valine, leucine, and isoleucine degradation, together with glycine, serine, and threonine metabolism, and fatty acid metabolism-related pathways were found.

These pathways point towards an increase in macromolecule synthesis. In addition, in lung and breast cancer, we observed enrichment and moderate impact in the phospholipase D signaling pathway, which is involved in many metabolic processes [76]. A more cancer-specific pathway, the central carbon metabolism in cancer pathway, was enriched, but was marginally impacted in lung and breast cancer. Lung cancer, on the other hand, was highly impacted in its glycolysis or gluconeogenesis pathway, a pathway critical for the growth of certain cancers, including lung cancer [77]. Alanine, aspartate, and glutamate metabolism was impacted moderately in various cancer types: breast, liver, and colorectal cancer. The latter also was impacted in the metabolism of the important antioxidant glutathione.

Besides metabolic deregulations, lung, colorectal, and breast cancer were enriched in multiple pathways that were related to cell adhesion and the cytoskeleton. We saw a substantial impact in the regulation of the actin cytoskeleton pathway and a very high impact in the focal adhesion pathway. Although mostly of little impact, we also detected enrichment in multiple inflammatory, pathogen, or immune response-related pathways in liver, and especially lung cancer. Furthermore, it became clear that both breast and lung cancer were strongly defined by the presence of various oncogene signaling and cancer-related pathways. We noticed in lung cancer that the mTORC1 and mTORC2 protein complexes, which play an important role in regulating cell growth in regard to nutrient availability [78], exhibit high overactivity, suggesting resistance to nutrient deprivation [79,80]. Another shared impacted pathway between lung and breast cancer was the EGFR tyrosine kinase inhibitor resistance pathway.

#### 2.4.2. Joint Pathway Analysis of Differentially Connected Genes and Metabolites

Parallel to joint pathway analysis using differential gene expression and metabolite abundance, a second joint pathway analysis was performed using the results from differential network analysis. While the results from differential metabolite network analysis proved useful, differentially connected genes did not. Since most cancer types yielded the same significant differentially connected genes, there is also high concordance between cancer types in the joint pathway analysis. These results therefore are deemed highly biased and will not be elaborated on (Figure 5B). PCA (Figure 5D) on the pathway impact scores revealed that breast cancer was mostly characterized by oncogene signaling pathways. Liver cancer, on the other hand, was more defined by its primary bile acid biosynthesis.

### 2.5. Cross-Talk between the Metabolomic and Transcriptomic Landscapes of Different Cancer Types

**Figure 6:**
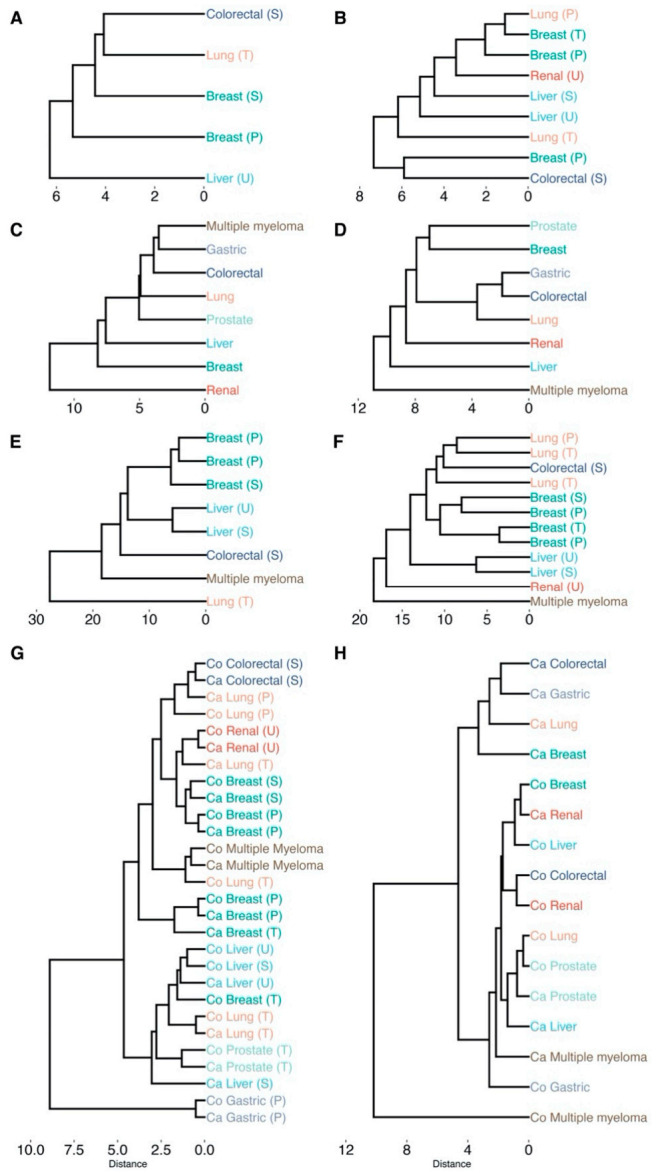
Hierarchical clustering of cancer types based on the impact on cellular pathways that are significantly enriched (*p_adj_* < 0.05). Labels are colored according to their cancer type. Sample origin is denoted as T, U, S, P for tissue, urine, serum and plasma, respectively (all transcriptomics analyses originate from tissue). All clustering is based on Euclidean distance and average linkage: (**A**) differentially abundant metabolites (*p_adj_* < 0.05); (**B**) differentially connected metabolites (*p_adj_* < 0.05); (**C**) differentially expressed genes (*p_adj_* < 0.05); (**D**) differentially connected genes (*p_adj_* < 0.05); (**E**) differentially abundant metabolites (*p_adj_* < 0.05) and differentially expressed genes (*p_adj_* < 0.05) conjoined; (**F**) differentially connected metabolites (*p_adj_* < 0.05) and differentially connected genes (*p_adj_* < 0.05) conjoined; (**G**) dendrogram based on the topological network measures per cancer type for all inferred metabolite association networks. Here, networks inferred from cancer or control samples are labeled as “Ca” and “Co”, respectively. (**H**) Dendrogram based on the topological network measures per cancer type for all inferred genes association networks. *p_adj_* indicates Benjamini–Hochberg corrected *p*-values.

The re-analysis of metabolomic and transcriptomic cancer data indicate that different cancer types are characterized by different metabolomic and transcriptomic landscapes and that different biological characteristics are highlighted when considering molecular signatures that are obtained using differentially abundant/connected metabolites and genes. The relationships between the different cancer types as expressed by differences and commonalities in the impacted and deregulated metabolic pathways are shown in the clustering trees in Figure 6A–F.

A high dissimilarity between cancer type clustering was obtained using different approaches (i.e., considering metabolomics of transcriptomics data and differentially abundant/expressed metabolites and genes) is observed. For instance, colorectal and lung cancer cluster together when considering differentially abundant metabolites (Figure 6A) but are dissimilar when considering differentially connected metabolites (Figure 6B). Multiple myeloma and gastric cancer share a similar transcriptomic landscape defined by differentially expressed genes (Figure 6C) but not when considering differentially connected genes (Figure 6D).

It is noteworthy that similar cancer types cluster together (Figure 6E,F) only when both metabolomic and transcriptomic data are considered indicating that cancer landscapes can be fully characterized only when working at different omics levels.

Although topological metrics of metabolite–metabolite association networks were not able to discriminate between controls and cancers, we see in Figure 6G that the same measures are efficient in capturing the similarities among the same cancer type. On the contrary, topological measures from gene–gene association networks (Figure 6H) can discriminate between cancer and controls but not among cancer types. This may well be an effect of different metabolite coverage: different experiments measure different panels of metabolites while transcriptomics experiments usually measure the same set of human genes.

**Figure 7:**
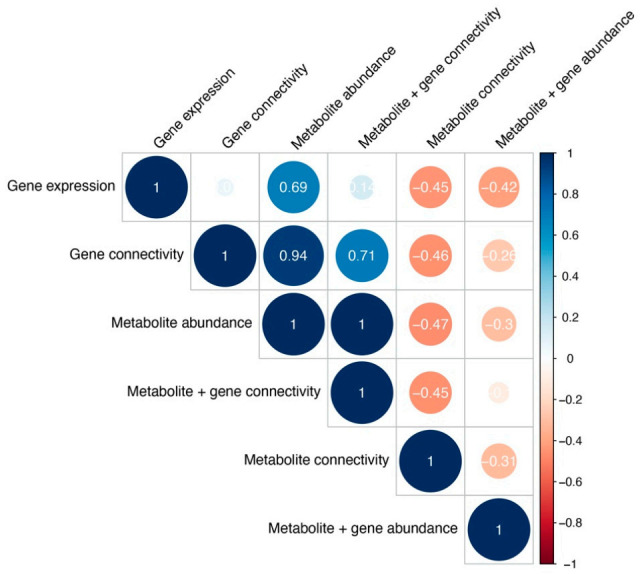
Cophenetic correlations between cancer type clustering across different analyses, measuring clustering similarity. Color and size represent the Cophenetic correlation coefficient, with negative correlations depicted as red and correlations as blue.

We quantified (diss)similarities in pathway impact of cancer types, by calculating the cophenetic correlation (*c_coph_*) between all pairs of clustering trees (see Methods): the corresponding correlation plot can be found in Figure 7. The correlations among the different clustering are mostly negative, indicating high dissimilarity between cancer type dendrograms of different approaches. However, we found agreement between cancer types based on metabolite abundance and metabolite connectivity and gene connectivity combined (*c_coph_* = 1). Furthermore, the likeliness of cancer types based on the pathway impact according to gene connectivity was similar to metabolite abundance (*c_coph_* = 0.94) and metabolite/gene connectivity joined (*c_coph_* = 0.71). Gene expression and metabolite abundance also agree on the kinship of different cancer types (*c_coph_* = 0.69).

## 3. Materials and Methods

### 3.1. Data Acquisition

#### 3.1.1. Inclusion Criteria for Cancer Metabolomics Data

To allow fair comparison and top-level data fusion, we defined a set of inclusion criteria that had to be met by both metabolomics and transcriptomics data sets to be included in our re-analysis:

1. Only contain human samples collected in a case–control setting.
2. Controls samples derived either from healthy subjects or from adjacent tissues from the same patient, histopathologically classified as non-cancerous.
3. Being derived from patient tissue, blood, or urine.
4. Patients should have not been treated with chemotherapy, radiation therapy, or small-molecule drugs at the time of sampling.
5. Contain a reasonable number of cancer and control samples (>5) and measured metabolites (>30).
6. Studies using cancer cell lines, xenografts, or organoids were not included.

#### 3.1.2. Metabolomics Data Collection

Two public repositories were searched for data from metabolomics-based cancer studies: the EMBL-EBI maintained MetaboLights database [81] (https://www.ebi.ac.uk/metabolights/ [82]) and the UC San Diego Metabolomics Workbench (https://www.metabolomicsworkbench.org/ [83]). Seven data sets from Metabolomics Workbench and zero from MetaboLights satisfied the inclusion criteria. Additionally, seven were obtained through literature search by either contacting the authors or from the supplementary material of the original studies. In total, 14 metabolomics cancer data sets were included. 

#### 3.1.3. Transcriptomics Data Collection

Transcriptomics data sets satisfying the inclusion criteria were downloaded from the GEO database [84] (https://www.ncbi.nlm.nih.gov/geo/ [85]). A total of 8 data sets were included in our re-analysis. 

#### 3.1.4. Data Processing and Standardization

All data sets (both metabolomics and transcriptomics) were formatted and stored as Rdata object (.rds). Cancer and control samples were put in separate data matrices, arranged in a feature × observation format, with the columns containing metabolite or gene names and the rows containing the sample ID. Samples from normal tissues and histopathological non-cancerous adjacent tissues were classified as normal/controls. Samples from different subtypes of the same cancer, i.e., fast- and slow-proliferating multiple myeloma were all classified cancer samples. Furthermore, metabolites or genes with more than 10% missing values were removed from the data set.

#### 3.1.5. Metabolite Names Standardization

Metabolite identifiers used in the original data sets (KEGG [86], HMDB [87], PubChem [88], Chebi [89] and Metlin [90] identifiers) were standardized to the canonical metabolite name as defined by the Human Metabolome Database [87] using the MetaboanalystR R package [75]. If different identifiers were used for the same metabolite and not all resulted in the same metabolite name, majority voting determines the chosen name. In the case that a canonical name could not be found, the name given by the authors was used instead. If metabolite identifiers occurred more than once in a data set, they were consolidated to a single instance by taking the mean over the different instances. Metabolite names that did not have a matching alternative name, were manually curated.

#### 3.1.6. Gene Names Standardization

All gene identifiers (names, Affymetrix probe names, etc.) in the downloaded transcriptomics data sets were converted to HGNC gene symbols and names [91,92] using the biomaRt R package [93]. If gene identifiers occurred more than once in a data set, they were consolidated to a single instance by taking the mean over the different instances.

#### 3.1.7. Missing Data Handling

Missing metabolite abundances/concentrations and gene expression values (if lower than 10% for a given metabolite/gene in each data set) were imputed using the impute R package [94] which uses nearest neighbor averaging with default parameters (number of neighbors *k* = 10).

### 3.2. Statistical Analysis

#### 3.2.1. Data Transformation

Metabolite abundances/concentrations and gene expression value were log-transformed before analysis.

#### 3.2.2. Metabolite and Gene Differential Abundance and Expression Analysis

Genes or metabolites that were differentially expressed or abundant were found using the EdgeR R package [95]. A linear model was fit to the data using least square regression. A contrast matrix was fit on this model to compute the estimated coefficients and standard errors comparing cancer and control groups. From this, the moderated *t*-statistic, *F*-statistic, and log-odds of differential values were computed using empirical Bayes.

#### 3.2.3. Correction for Multiple Testing

*p*-Values for metabolite were adjusted using the Benjamini–Hochberg multiple testing correction [96]. Due to the high number of genes, the stricter Bonferroni family-wise error rate was used for all transcriptomics analyses. Genes or metabolites with an adjusted *p*-value < 0.05 were considered differentially expressed or abundant at the α = 0.05 significance level.

#### 3.2.4. Multivariate Analysis

Principal Component Analysis was used to visualize and explore high-dimensional representations of each data set in terms of biological pathways (see Section 2 for more details). Data were scaled to unit variance before analysis.

#### 3.2.5. Clustering

To determine similarities between cancer types, hierarchical clustering was used [97]. The Euclidean distance between the first three principal components of each cancer type was clustered with average linkage.

#### 3.2.6. Comparing Cancer Type Clustering

The clustering of cancer types was compared between different analyses. As only dendrograms with the same structure can be considered for direct comparison, leaves of dendrograms were pruned to produce intersecting trees in a pairwise manner. If multiples of the same cancer type were present in a dendrogram, the centroid was used to create the initial clustering before pruning. To quantify the difference between the two dendrograms, the cophenetic correlation was calculated which is defined as the intergroup dissimilarity at which two observations in each dendrogram are first combined into a single cluster [98].

#### 3.2.7. Network Inference

To infer metabolite–metabolite and gene–gene association networks, we used a modified implementation of the Probabilistic Context Likelihood of Relatedness based on Correlations (PCLRC) [99]; standard correlations between two molecular features *i* and *j* (either metabolites or genes) where replaced with partial correlations obtained using a Gaussian Graphical Model (GMM). The PCLRC algorithm uses resampling to estimate robust correlation based on the Context Likelihood of Relatedness approach [100] which estimates the relevance of the associations between two features by considering background associations. The PCLCR returns a probability matrix **P**, containing the likelihood 0 < *p_ij_* < 1 of each observed association *r_ij_* between each metabolite or gene pair. Significant associations were defined as

(1) $$r_{ij}=\;\left\{\begin{array}{c} r_{ij}\;\;\;\;\;\;\;if\;p_{ij}\geq 0.95\\ 0\;\;\;\;\;\;\;\;\;if\;p_{ij}<0.95 \end{array}\right..$$

Default parameters were used (number of resampling iterations *Niter* = 1000; the fraction of the samples to be considered at each iteration *frac* = 0.75 and fraction of the total predicted interactions to be kept at each iteration *rank.thr* = 0.3.). All networks are undirected and represented as an adjacency matrix *M*, populated by interactions (edges) between metabolite/gene *i*,*j* (nodes).

#### 3.2.8. Estimation of Partial Correlations Using Gaussian Graphical Models

Partial correlations were estimated using a Gaussian Graphical Model as implemented in the GeneNet R package [101,102]. GeneNet allows estimating a GGM from a small sample of high-dimensional data in a computationally and statistically efficient way. It uses an analytic shrinkage estimation of covariance and partial correlation matrices and performs optimal model selection based on local false discovery rate multiple testing. The edges (i.e., the associations) to be included in the final association network are selected using a computational algorithm depending on the relative values of the pairwise partial correlations. For more details on GeneNet implementation, we refer to the original publication. Networks were inferred for both the transcriptomics and metabolomics data sets. For each data set, separate interaction networks were computed for cancer and control groups, respectively. To reduce noise and computational load of the network estimation from transcriptomics data, only the most variable genes were considered. These were defined as genes that occur in all transcriptomics data sets and have a variance that is one standard deviation higher than the mean variance across all data sets. To make this one sigma cutoff, variances for all genes were log-transformed to normalize them before calculating the mean and standard deviation of the variance. Furthermore, for genetic association networks, *Niter* = 100, *MaxPerm* = 100, and a probability threshold of 0.99 were used.

#### 3.2.9. Differential Node Connectivity

The connectivity each node *i* in a given association network (either specific to cancer or control samples), corresponding to either a gene or metabolite, is defined as:

(2) $$\chi_{i}=\;\left(\sum_{j=1}^{J}\left|r_{ij}\right|\right)-1.$$

The differential connectivity Δ*_i_* for metabolites or genes *i* is defined as subtracting the connectivity of control group from the cancer group:

(3) $$\Delta_{i}^{\mathrm{cancer},\;\mathrm{control}}=\;\left|\chi_{i}^{cancer}-\chi_{i}^{control}\right|$$

#### 3.2.10. Assessment of the Significance of Differential Connectivity

Statistical significance of these differentially connected nodes (metabolites/genes) was assessed using a permutation test. Briefly, the columns of input data matrices were independently permuted to destroy the relationship among variables while preserving their variance obtaining a permuted matrix M_k_. For each node *i* in the permuted matrix M_k_, the differential connectivity was determined as:

(4) $$\Delta_{i,k}^{\mathrm{cancer},\;\mathrm{control}}=\chi_{i,k}^{cancer}-\chi_{i,k}^{control}$$

Repeatedly permutating for *k* = 1000 yields an empirical null distribution *D_i_* of differential connectivity values Δ*_i,k_*. For each node in the network. From *D_i_*, the corresponding *p*-value for Δ*_i_* (for the original, non-permuted network connectivity of node *i*) is calculated as:

(5) $$p_{\Delta_{i}}=\frac{1+\left(\left|D_{i}\right|>|\Delta_{i}^{cancer,\;\;\;control}|\right)}{k}.$$

*p*-values for differentially connected metabolite and genes were corrected for multiple testing using the Benjamini–Hochberg method.

#### 3.2.11. Metrics for Network Topology Characterization

The topology of interaction networks was characterized using various metrics besides node connectivity 𝜒*_i_*. We used *Centralization*, *Diameter*, *Hub Nodes*, *Betweenness*, *Closeness*, *Degree*, *Minimal Distance*, *Page rank* and *Transivity*. All topological measures considered were calculated using the Igraph R package [103] and are defined in Appendix B.

#### 3.2.12. Software

All code used for analysis is available in the Supplementary File Code.zip.

## 4. Conclusions

In this study, we attempted a joined re-analysis of publicly available metabolomics and transcriptomics data to examine the metabolic and gene expression profiles of different cancer types and to investigate similarities and dissimilarities that can be described at different omics levels. Despite living in the era of Open Access to information, knowledge, and data, metabolomics cancer data sets proved difficult to acquire. 

The metabolomic community has proposed and agreed on data standards and reporting guidelines [104,105] but has not yet embraced broad data science. In contrast, the genomics community has strong precedents for broad data sharing and open science, most notably the Bermuda Principles of 1996 [106]: since then, many scientific journals have made deposition of transcriptomics profiles to the GEO database as (almost) a pre-condition for publication. In fact, and we found no difficulties in finding and accessing high-quality cancer transcriptomics data satisfying our inclusion criteria and matching the selected metabolomics data in terms of cancer types. A comprehensive re-analysis of cancer metabolomics data sets is challenging at least. Low-level data fusion (i.e., merging of data sets before analysis) [107,108] of publicly available data sets, when obtained, is challenged by the different number and type of metabolites measured and different sample sizes. At the moment, the only viable solution for the de novo re-analysis of existing metabolomics cancer data is to perform high-level data fusion, i.e., applying the same analysis on different data sets and then integrate and compare the results as in the present study.

Metabolomic and transcriptomic landscapes highlight different patterns of variability among different cancer types to the point that some cancers are similar in the metabolomic space but not in the transcriptomic space and vice versa. This can be attributed to the fact that the fluxes of metabolically regulated reactions are mainly a function of the substrates and product levels, while the fluxes of transcriptionally regulated reactions are mainly controlled by the expression level of the catalyzing enzymes [109]. Such differences may also be attributed to different metabolite coverages among different experimental studies, indicating the necessity of standardized metabolomics experiments to directly compare different cancer types. Moreover, it proved to be impossible to obtain data where metabolomics and transcriptomics data were obtained from the same patients. This adds heterogeneity to the data that is difficult to overcome.

Overall, our results confirm that deregulations of protein metabolism and cell cycle pathways are the main hallmarks of cancer, but cancer-specific signatures exist that are better captured when both metabolite and gene expression landscapes are considered simultaneously. For instance, cell cycle was mainly impacted in breast, liver, and colorectal cancer, while liver cancer was found to be impacted in a relatively large number of metabolic pathways, but especially in valine, leucine, and isoleucine degradation. Breast and lung cancer both showed aberrations in cell adhesion mechanisms and several oncogene signaling-related pathways. We also showed the added value of using topological features of association networks representing molecular codependences that are able to accurately distinguish between cancer and control cases in genetic networks. 

In summary, there is a great benefit in integrating and analyzing data at different omics levels to elucidate the differences and similarities of cancer landscapes. However, a true fusion of metabolomics and transcriptomics can only be accomplished through improved metabolomic data reporting and sharing.

## Figures and Tables

**Table 1:** A summary of all 14 metabolomics and 8 transcriptomics data sets across eight different cancer types and different sampling origins. Data sets that are not accompanied by a paper are provided with their corresponding database ID from either the Metabolomics Workbench or the Gene Expression Omnibus.

PubMed ID	Database ID	Data Type	Cancer Type	Sample Type	Cancer Samples	Control Samples	Metabolites/Genes
	ST000054	Metabolomics	Breast	Tissue	121	23	65
27036109 [15]	ST000355	Metabolomics	Breast	Plasma	138	77	221
27036109 [15]	ST000356	Metabolomics	Breast	Serum	104	32	262
31794572 [16]		Metabolomics	Breast	Plasma	80	60	69
25126899 [17]	ST000284	Metabolomics	Colorectal	Serum	66	92	113
29259332 [18]		Metabolomics	Gastric	Plasma	19	20	107
21518826 [19]		Metabolomics	Liver	Serum	82	71	66
21518826 [19]		Metabolomics	Liver	Urine	82	71	77
	ST000390	Metabolomics	Lung	Tissue	70	10	182
	ST000396	Metabolomics	Lung	Plasma	41	200	126
23543897 [20]		Metabolomics	Lung	Tissue	9	9	92
	ST000221	Metabolomics	Multiple myeloma	Tissue	9	6	191
23543897 [20]		Metabolomics	Prostate	Tissue	7	7	72
21348635 [21]		Metabolomics	Renal	Urine	25	25	205
31594947 [22]	E-MTAB-6703	Transcriptomics	Breast	Tissue	2088	214	20,545
31594947 [22]	E-MTAB-6698	Transcriptomics	Colorectal	Tissue	1393	121	20,545
31594947 [22]	E-MTAB-6693	Transcriptomics	Gastric	Tissue	691	46	20,107
31594947 [22]	E-MTAB-6695	Transcriptomics	Liver	Tissue	264	137	20,107
31594947 [22]	E-MTAB-6699	Transcriptomics	Lung	Tissue	1474	147	20,545
24816239 [23]	GSE47552	Transcriptomics	Multiple myeloma	Tissue	41	4	22,470
31594947 [22]	E-MTAB-6694	Transcriptomics	Prostate	Tissue	121	116	20,107
31594947 [22]	E-MTAB-6692	Transcriptomics	Renal	Tissue	219	104	20,107

**Table 2:** Overview of the number of significantly differentially abundant metabolites per data set and cancer type (Benjamini–Hochberg adjusted *p*-value *p_adj_* < 0.05).

Data Set	Cancer	Type	Metabolites	Differentially Abundant (*p_adj_* < 0.05)
31794572 [16]	Breast	Plasma	69	41
ST000054	Breast	Tissue	65	2
27036109 [15]	Breast	Plasma	221	87
27036109 [15]	Breast	Serum	262	182
25126899 [17]	Colorectal	Serum	113	25
29259332 [18]	Gastric	Plasma	104	4
21518826 [19]	Liver	Serum	63	30
21518826 [19]	Liver	Urine	77	47
23543897 [20]	Lung	Tissue	92	43
ST000396	Lung	Plasma	126	1
ST000390	Lung	Tissue	182	3
ST000221	Multiple myeloma	Tissue	191	114
23543897 [20]	Prostate	Tissue	72	0
21348635 [21]	Renal	Urine	205	0

**Table 3:** Topological network characteristics of cancer (Ca) and control (Co) metabolite association networks across cancer types. Sample origin is labeled as T, U, S, P for tissue, urine, serum, and plasma, respectively. MM is multiple myeloma and data set identifiers are noted as: * ST000054, ^§^ ST000221, ^#^ ST000390 and ^†^ ST000396. Topological measures in the column names are: N = Nodes, E = Edges, Mean Conn. = Mean Connectivity, Mean Deg. = Mean Degree, Mean Close. = Mean Closeness, Mean Betw. = Mean Betweenness, D = Diameter, Mean Min. Dist. = Mean Minimal Distance, Mean Page Rank, Hub N = Hub Nodes, Centr. = Centralization, and Trans. = Transivity. All topological measures are further explained in the material and methods.

Network	N	E	Mean Conn.	Mean Deg.	Mean Close.	Mean Betw.	D	Mean Min. Dist.	Mean Page Rank	Hub N	Centr.	Trans.
Ca Renal (U) [21]	203	1939	0.407	0.095	0.348	0.009	6	2.886	0.005	95	0.163	0.191
Co Renal (U) [21]	203	1889	0.435	0.092	0.344	0.010	5	2.920	0.005	100	0.225	0.195
Ca Liver (S) [19]	62	229	0.547	0.121	0.205	0.067	11	5.003	0.016	43	0.207	0.036
Co Liver (S) [19]	62	191	0.428	0.101	0.051	0.036	11	4.489	0.016	40	0.129	0.025
Ca Liver (U) [19]	77	357	0.373	0.122	0.081	0.028	9	3.588	0.013	46	0.220	0.247
Co Liver (U) [19]	77	289	0.355	0.099	0.076	0.036	10	4.394	0.013	40	0.164	0.149
Ca Lung (T) [20]	91	632	0.272	0.154	0.152	0.026	8	3.484	0.011	14	0.223	0.457
Co Lung (T) [20]	91	612	0.263	0.149	0.135	0.023	8	3.239	0.011	15	0.295	0.372
Ca Prostate (T) [20]	72	294	0.168	0.115	0.052	0.045	16	5.513	0.014	9	0.167	0.447
Co Prostate (T) [20]	72	276	0.148	0.108	0.065	0.049	12	5.493	0.014	21	0.117	0.482
Ca Gastric (P) [18]	103	10204	2.072	1.943	0.243	0.000	1	1.000	0.010	0	2.018	1.000
Co Gastric (P) [18]	103	10004	2.225	1.904	0.193	0.000	1	1.000	0.010	0	2.017	1.000
Ca Breast (P) [16]	68	585	1.094	0.257	0.436	0.020	4	2.307	0.015	39	0.131	0.157
Co Breast (P) [16]	68	637	1.078	0.280	0.449	0.019	4	2.238	0.015	28	0.168	0.185
Ca Breast (T) *	65	499	0.566	0.240	0.370	0.028	5	2.750	0.015	8	0.229	0.334
Co Breast (T) *	65	293	0.409	0.141	0.106	0.042	10	4.130	0.015	23	0.234	0.397
Ca MM (T) ^§^	191	3171	0.233	0.175	0.334	0.011	6	3.073	0.005	13	0.404	0.613
Co MM (T) ^§^	191	2807	0.217	0.155	0.328	0.011	6	3.065	0.005	1	0.151	0.506
Ca Colorectal (S) [17]	113	813	0.650	0.128	0.347	0.017	6	2.911	0.009	59	0.122	0.169
Co Colorectal (S) [17]	113	713	0.710	0.113	0.327	0.019	7	3.100	0.009	69	0.084	0.141
Ca Breast (P) [15]	221	2863	0.941	0.118	0.389	0.007	4	2.585	0.005	78	0.146	0.215
Co Breast (P) [15]	221	2857	0.853	0.118	0.389	0.007	5	2.581	0.005	78	0.110	0.224
Ca Breast (S) [15]	260	3778	0.667	0.112	0.390	0.006	5	2.581	0.004	89	0.228	0.215
Co Breast (S) [15]	260	3500	0.463	0.104	0.377	0.006	6	2.675	0.004	69	0.143	0.233
Ca Lung (T) ^#^	182	1614	0.732	0.098	0.261	0.010	5	2.831	0.005	123	0.101	0.153
Co Lung (T) ^#^	182	2116	0.274	0.128	0.258	0.010	6	2.885	0.005	19	0.225	0.398
Ca Lung (P) ^†^	126	878	0.460	0.111	0.247	0.016	7	3.055	0.008	62	0.113	0.189
Co Lung (P) ^†^	126	914	0.833	0.116	0.169	0.014	5	2.844	0.008	98	0.140	0.110

**Table 4:** Overview of the number of significantly differentially connected metabolites per data set and cancer type (Benjamini–Hochberg adjusted *p*-value *p_adj_* < 0.05) according to inferred metabolite association networks.

Data Set	Cancer	Type	Metabolites	Differentially Connected
31794572 [16]	Breast	Plasma	69	60
ST000054	Breast	Tissue	65	48
27036109 [15]	Breast	Plasma	221	181
27036109 [15]	Breast	Serum	262	191
25126899 [17]	Colorectal	Serum	113	100
29259332 [18]	Gastric	Plasma	104	4
21518826 [19]	Liver	Serum	63	52
21518826 [19]	Liver	Urine	77	64
23543897 [20]	Lung	Tissue	92	13
ST000396	Lung	Plasma	126	114
ST000390	Lung	Tissue	182	139
ST000221	Multiple myeloma	Tissue	191	48
23543897 [20]	Prostate	Tissue	72	0
21348635 [21]	Renal	Urine	205	109

**Table 5:** Overview of the number of significantly differentially expressed genes per cancer type (Benjamini–Hochberg adjusted *p*-value (*p_adj_*) < 0.05).

Data Set	Cancer	Genes	Differentially Expressed
E-MTAB-6703 [22]	Breast	20,545	2892
E-MTAB-6698 [22]	Colorectal	20,545	5577
E-MTAB-6693 [22]	Gastric	20,107	691
E-MTAB-6695 [22]	Liver	20,107	5225
E-MTAB-6699 [22]	Lung	20,545	9086
GSE47552 [23]	Multiple myeloma	22,470	132
E-MTAB-6694 [22]	Prostate	20,107	3691
E-MTAB-6692 [22]	Renal	20,107	9265

**Table 6:** Topological network characteristics of cancer (Ca) and control (Co) gene association networks across cancer types. Multiple myeloma is abbreviated as MM. Topological measures in the column names are: N = Nodes, E = Edges, Mean Conn. = Mean Connectivity, Mean Deg. = Mean Degree, Mean Close. = Mean Closeness, Mean Betw. = Mean Betweenness, D = Diameter, Mean Min. Dist. = Mean Minimal Distance, Mean Page Rank, Hub N = Hub Nodes, Centr. = Centralization, and Trans. = Transivity. All topological measures are further explained in the material and methods.

Network	N	E	Mean Conn.	Mean Deg.	Mean Close.	Mean Betw.	D	Mean Min. Dist.	Mean Page Rank	Hub N	Centr.	Trans.
Ca Renal [22]	2570	148,296	0.811	0.045	0.433	0.001	3	2.311	0.000	2347	0.040	0.082
Co Renal [22]	2570	164,272	0.576	0.050	0.440	0.000	3	2.277	0.000	2055	0.086	0.121
Ca Gastric [22]	2570	111,026	1.273	0.034	0.398	0.001	4	2.513	0.000	2349	0.040	0.063
Co Gastric [22]	2570	163,932	0.366	0.050	0.425	0.001	4	2.357	0.000	1508	0.087	0.209
Ca Prostate [22]	2570	160,446	0.627	0.049	0.442	0.000	3	2.267	0.000	2251	0.061	0.091
Co Prostate [22]	2570	166,152	0.629	0.050	0.443	0.000	3	2.262	0.000	2149	0.078	0.107
Ca Liver [22]	2570	156,934	0.922	0.048	0.441	0.000	3	2.270	0.000	2358	0.047	0.081
Co Liver [22]	2570	151,116	0.620	0.046	0.433	0.001	3	2.314	0.000	2228	0.061	0.090
Ca Colorectal [22]	2570	108,138	1.687	0.033	0.398	0.001	4	2.518	0.000	2484	0.033	0.041
Co Colorectal [22]	2570	179,636	0.666	0.054	0.449	0.000	3	2.232	0.000	1904	0.074	0.139
Ca Lung [22]	2570	80,344	1.440	0.024	0.371	0.001	4	2.695	0.000	2483	0.030	0.038
Co Lung [22]	2570	168,414	0.713	0.051	0.443	0.000	3	2.264	0.000	2063	0.073	0.117
Ca Breast [22]	2570	50,256	1.164	0.015	0.334	0.001	5	3.000	0.000	2512	0.018	0.034
Co Breast [22]	2570	155,668	0.812	0.047	0.438	0.001	3	2.286	0.000	2283	0.050	0.082
Ca MM [23]	2570	145,712	0.325	0.044	0.418	0.001	3	2.395	0.000	2067	0.087	0.158
Co MM [23]	2570	356,528	0.152	0.108	0.292	0.001	6	3.423	0.000	0	0.080	0.602

**Table 7:** Overview of the number of significantly differentially expressed genes per cancer type (*p_adj_* < 0.05) according to inferred gene association networks. *p_adj_* indicates Benjamini–Hochberg corrected *p*-values.

Data Set	Cancer	Genes	Differentially Connected (*p_adj_* < 0.05)
E-MTAB-6703 [22]	Breast	2570	2404
E-MTAB-6698 [22]	Colorectal	2570	2550
E-MTAB-6693 [22]	Gastric	2570	2552
E-MTAB-6695 [22]	Liver	2570	2345
E-MTAB-6699 [22]	Lung	2570	2536
GSE47552 [23]	Multiple myeloma	2570	2005
E-MTAB-6694 [22]	Prostate	2570	2146
E-MTAB-6692 [22]	Renal	2570	2155

## Data Availability

Transcriptomic data used in this study is available at http://doi.org/10.5281/zenodo.4314604.

## Supplementary Materials

The following are available online at https://www.mdpi.com/2072-6694/13/3/393/s1, Table S1: Metabolite occurrence across all data sets; Code.zip: Computer code (R 3.6.3) used for analysis; Data.zip: Standardized metabolomic data sets and selected genes; Pathway.zip: results of pathway analysis.

## Appendix A

Through manual curation of metabolic profiling studies, we selected 220 papers adhering to our inclusion criteria. From these papers, 11 provided direct access to the data. After contacting 129 corresponding authors, we managed to acquire three more. In total, we considered 14 data sets encompassing eight different cancer types: breast, lung, gastric, renal, liver, colorectal, prostate, and multiple myeloma and contains samples from organ tissue (five), urine (two), and blood (four plasma, three serum) as given in Table 1 together with data descriptors. After cleaning and standardization, the data sets comprised of 1095 different metabolites suitable for analysis. The most ubiquitous metabolite is threonine, followed by creatinine, phenylalanine, proline, valine, and pyroglutamic acid, occurring in nearly all data sets. A complete list of all metabolite occurrences can be found in Supplementary Table S1.

For each of the eight different cancer types represented by the 14 metabolomics data sets, we were able to find and access high-quality cancer transcriptomics data satisfying our inclusion criteria and matching the selected metabolomics data in terms of cancer types. Seven out of eight data sets originate from a homogenized microarray compendium consisting of 95 different GEO studies [22]. A list of the cancer data sets obtained, and associated data descriptors is given in Table 1**.** The eight data sets cover 22,470 genes of which 16,040 are common among all data sets.

## Appendix B

Inferred association networks are defined as a matrix *M_ij_,* where *i* and *j* are nodes. In this matrix, the number of connections node *i* has, is defined by its *degree k*, where

(A1) $$k_{i}=\frac{\sum_{j}m_{ij}}{N-1}$$

with *N* being the number of nodes in a network, which is used no normalize for network size. Size normalization allows for direct comparison between differently sized networks. Node degree is equal to connectivity in binary networks.

Similar to node degree, *Closeness* was determined, which measures the distance of a node to all other nodes. Closeness is defined as:

(A2) $$C_{i}=\;\frac{1}{\frac{\sum_{j}d_{ij}}{N-1}}$$

where *d_ij_* is the distance between nodes *i* and *j* [110].

Another centrality measure, betweenness, represents the extent to which nodes are in-between each other and is defined as:

(A3) $$B_{i}=\sum_{v\neq j,v\neq i,j\neq i}\frac{\sigma_{vij}}{\sigma_{vj}},$$

where *𝜎_vj_* is the total number of shortest paths from node *v* to *j* and *𝜎_vij_* is the number of those paths passing through node *i*. Node betweenness is also normalized for network size by:

(A4) $$B_{i\;norm}=\frac{2*B_{i}}{N\left(N-3\right)\left(N-2\right)}$$

In addition, the importance of a node was also described by its page rank [111] and whether it was a hub node. Page rank was calculated using the PRPACK algorithm (https://github.com/dgleich/prpack) [112] using 0.85 as damping factor. The page rank of a node is calculated as:

(A5) $$x_{i}=\sum_{j\in L_{i}}\frac{x_{j}}{n_{j}},$$

where *L_i_* is the set of nodes that link to node *i* and *n_j_* is the number of nodes that are liked to by node *j*. A node *i* was considered a hub node, on the other hand, if it has a clustering coefficient *c_i_ < 0.03* and a degree *k_i_ > 5* [113], where:

(A6) $$c_{i}=\frac{number\;of\;triangles\;connected\;to\;node\;i}{number\;of\;triples\;centered\;around\;node\;i},$$

with a triple centered around node *i* being defined as a set of two edges connected to *i*.

Since different networks generally do not contain identical nodes, the mean of all node-level topology measures was taken to compare the characteristics of whole networks. However, the topology of each network was also described using multiple global measures. The size of a network was described by the number of nodes and the number of edges $\sum m_{ij}$. Additionally, the diameter *D* and the mean shortest path length ℓ of the network was computed, where:

(A7) $$D=\max\left(s_{ij}\right),$$

and

(A8) $$\ell =\frac{1}{N\left(N-1\right)}\sum_{i\neq j}s_{ij},$$

with *s_ij_* being the number of edges in the shortest path from node *i* to *j.*

Besides network size, its shape was evaluated by measuring a network level centralization metric using degree centrality:

(A9) $$C_{M}=\frac{\sum_{i}(\max\left(k_{i}\right)-k_{i})}{N^{2}-3N+2},$$

where a *C_M_* of 1 would mean a star network and a *C_M_* of 0 a fully connected network.

Finally, the tendency of a network to exhibit modular organization can be measured by the transivity, which is a global clustering coefficient [114,115]:

(A10) $$c=\frac{1}{N}\sum_{i}C_{i}.$$
